# Meteorological Journal

**Published:** 1824-01

**Authors:** 


					t 86 J
METEOROLOGICAL JOURNAL,
From November 20, to December 19, 1823.
By Messrs. William Harris and Co. 50, Holborn, London.
Nov
20
21
25
26
27
30
Dec
1
2
3
4
5
6
7
8
9
10
11
12
13
14
15
16
17
18
19
Rain
gauge
.73
_4S_
118
.25
.35
29.94
30.05
29.86
29.90
29.92
30.10
30.10
30.07
29.84
29.60
29.35
29.60
29.43
29.44
29.27
29.70
29.37
30.35
30.30
30.22'
30.28
30.00
29.70
29.80
30.10
30.04
30.05
29.57
29.34
29.50
30.03
30.00
29.80
29.90
30.00
30.13
30.10
30.00
29.84
29.40
29.40
29.77
29.36
29.38
29.47
29.60
29.83
30.38
30.20
30.25
30.20
29.93
29.67
29.96
30-02
30.14
30.00
28.85
39.40
29,62
DE
LUC'S
HYG.
9 A.M
sw
SW
ssw
s
s
sw
sw
s
s
s
s
sw
s
sw
sw
sw
N
w,
wsw
sw
sw
s
sw
WNW
w
w
s
s
sw
NNW
10P.M
sw
sw
s
ssw
ssw
wsw
wsw
s
s
s
s
sw
s
s
sw
ssw
NNW
wsw
wsw
wsw
sw
ssw
wsw
NW
ssw
wsw
sw
S var.
SW
sw
ATMOSPHERIC
VARIATION.
9 A.M,
Overc.
Cloud.
Cloud.
Foggy
Cloud.
Rain
Fine
Cloud.
Rain
Fine
Faii-
Raia "ain
2 P.M.
Fair
Fine
Fair
Cloud,
Fail-
Cloud,
Rain
Cloud.
Rain
Fine
Foggy
Overc.
Fine
Fine
Foggy
Fine
Rain
Fine
Cloud.
Fine
Foggy
Fine
Fair
Fine
Rain
Fine
10p.m.
Fair
Fine
Cloud.
Fair
Rain
Fine
Cloud.
Rain
Fine
Cloud.
Cloud.
Foggy
Fair
Fine
Foggy
Fine
Rain
Storm.
Cloud.
Cloud.
The quantity of rain fallen in the month of November,
was 1 inch and 18.100ths.

				

